# Thermal stabilization of enterovirus A 71 and production of antigenically stabilized empty capsids

**DOI:** 10.1099/jgv.0.001771

**Published:** 2022-08-23

**Authors:** Natalie J. Kingston, Mona Shegdar, Joseph S. Snowden, Helen Fox, Elisabetta Groppelli, Andrew Macadam, David J. Rowlands, Nicola J. Stonehouse

**Affiliations:** ^1^​ Astbury Centre for Structural Molecular Biology, School of Molecular and Cellular Biology, Faculty of Biological Sciences, University of Leeds, Leeds, UK; ^2^​ Division of Virology, National Institute for Biological Standards and Control, Potters Bar, Hertfordshire, UK; ^‡^​ Present address: Institute for Infection and Immunity, St George’s University of London, Tooting, London, UK

**Keywords:** EVA71, empty capsids, stabilization, infantile paralysis

## Abstract

Enterovirus A71 (EVA71) infection can result in paralysis and may be fatal. In common with other picornaviruses, empty capsids are produced alongside infectious virions during the viral lifecycle. These empty capsids are antigenically indistinguishable from infectious virus, but at moderate temperatures they are converted to an expanded conformation. In the closely related poliovirus, native and expanded antigenic forms of particle have different long-term protective efficacies when used as vaccines. The native form provides long-lived protective immunity, while expanded capsids fail to generate immunological protection. Whether this is true for EVA71 remains to be determined. Here, we selected an antigenically stable EVA71 virus population using successive rounds of heating and passage and characterized the antigenic conversion of both virions and empty capsids. The mutations identified within the heated passaged virus were dispersed across the capsid, including at key sites associated with particle expansion. The data presented here indicate that the mutant sequence may be a useful resource to address the importance of antigenic conformation in EVA71 vaccines.

## Introduction

Enteroviruses (EVs) are globally distributed and are associated with a range of pathologies ranging from the common cold (human rhinovirus; HRV) to lethal neurological disease (poliovirus; PV). Enterovirus A 71 (EVA71) is the primary causative agent of hand, foot and mouth disease (HFMD), a generally mild, self-limiting condition affecting young children. In severe cases the disease can present with neurological complications reminiscent of poliomyelitis [1–4]. The financial and humanitarian cost associated with HFMD has encouraged the development of a number of vaccines against EVA71. Subunit, DNA and virus-like particle (VLP) vaccines have all been investigated in murine models and each showed the ability to induce protection against lethal challenge [5–10]. The most advanced vaccine approach is based upon inactivated virus, and these vaccines have been licensed in PR China, where EVA71 is responsible for periodic outbreaks of HFMD [11–15]. In PR China, the introduction of two-dose vaccination of children aged 6–59 months in December 2015 resulted in an overall reduction in disease and lower rates of serious or fatal infection [16–18].

While the ability of inactivated virus vaccines to control the spread of EVA71 is extremely promising, there remains a significant biosafety concern associated with the large-scale production of infectious virus required for the generation of inactivated virus vaccines. There have been several instances where the production of PV vaccine material has resulted in the accidental release of infectious virus. In 1995 the incomplete inactivation of PV in ≈120 000 vaccine doses resulted in ≈40 000 cases of muscle weakness in recipients, 51 individuals were paralysed and 5 died. Additionally, spread of the virus to close contacts of the vaccine recipients resulted in an additional 113 cases and 5 deaths [19, 20]. Between 1991–1993 the vaccine strain of PV type 3 (Saukett) and PV type 1 (Mahoney) were isolated from multiple individuals. Several years later, the previously eradicated, PV type 2 vaccine strain (MEF) was isolated in India. In 2014, an incident in Belgium resulted in accidental release of infectious PV type 3 (Saukett) into the sewage system [21–24]. All of these instances occurred after the global PV eradication initiative was announced in 1988. The development of VLP vaccines would remove safety concerns associated with the large-scale production of infectious virus.

EV VLPs are composed of three viral structural proteins, VP0, VP3 and VP1, assembled as icosahedral capsids that lack infectious genetic material, rendering these particles inherently safe [25–28]. EV virion assembly occurs when the viral structural proteins package the genome, inducing an autocatalytic event in which VP0 is cleaved into VP4 and VP2 to generate the mature infectious virion [29]. Genome packaging and VP0 cleavage initiates the reorganization of the internal network of capsid proteins, which increases the stability of the infectious ‘native’ (NAg) conformation. Empty capsids (ECs) are also produced during infection and are similar to VLPs in that they are composed of structural proteins and lack viral genetic material. Nascent VLPs and ECs are antigenically indistinguishable from infectious virus, although at moderate temperatures they are converted to an expanded conformation (HAg), which is structurally similar to the virus in the post-receptor engaged state [29–34]. The immunological consequences of antigenic conversions are relatively well understood for PV, where only NAg particles elicit long-lived protective responses. Whether this holds true for EVA71 remains to be determined.

Here, we selected an antigenically stable EVA71 virus population using successive rounds of heating and passage and characterized the antigenic conversion of the virion and ECs. The mutant described will be a useful resource to address the importance of antigenic conformation in EVA71 vaccines and could inform the design of stabilized EVA71 VLPs for future vaccine production.

## Methods

### Cells and viruses

EVA71 strain MS/7423/87 was a gift from SingVax (Singapore); an EVA71 reverse genetics system was generated from this strain as previously described [35]. HeLa cells were obtained from the National Institute of Biological Standards and Control (NIBSC) and L929 and Vero cells were obtained from ATCC.

### Thermal selection of virus

To select thermally resistant virus, and similar to previously described protocols [36], 100 µl of 2×10^6^ p.f.u. ml^−1^ virus was incubated at 52.5 °C for 30 min and an aliquot stored on ice as a non-heated control. After incubation at a range of elevated temperatures the samples were directly titrated by plaque assay and inoculated into T25 flasks of confluent Vero cells. These were incubated at 37 °C until full cytopathic effect (CPE) was observed. Supernatants were clarified and used for subsequent cycles of heating and passage until the titres obtained from the unheated and heated virus populations were similar for at least three consecutive passages. This resulted in a total of nine cycles of heating and passaging. Samples from passages 4, 5, 7, 8 and 9 were analysed by next-generation sequencing (NGS). The virus isolated after nine passages with heating was subjected to five further passages in the absence of heating to assess the genetic stability of the thermally resistant virus population.

### Next-generation sequencing

The genetic sequence of stabilized virus populations was determined by next-generation sequencing using previously described protocols [37]. Briefly, RNA was extracted from virus samples and genomes were reverse transcribed, and then amplified cDNA was sequenced using a MiSeq flow cell (Illumina). Reads underwent quality trimming and assembly before mapping to parental reference sequences using Geneious R7 (Biomatters) software. Single nucleotide polymorphism (SNPs) present at 0.5 % or greater were identified (Tables 1, S1 and S2, available in the online version of this article).

**Table 1.** Frequency of non-synonymous mutations within the P1 region*.* Summary of the emergence of non-synonymous mutations within the structural region of virus. NGS was carried out, and mutation frequency is represented as a percentage of total reads at a given site

	VP2 V85L	VP3 I235M	VP1 Y116C	VP1 K162I	VP1 P246A
Untreated	−	−	−	−	+
Passage 4	24.3	95.5	2.3	15.6	95.2
Passage 5	14.5	97.2	2.3	21.2	97.2
Passage 7	9.2	100	22.8	99.6	100
Passage 8	20.6	99.6	43.5	99.9	99.7
Passage 9	43.1	99.7	69.5	99.9	99.7

### Generation of an infectious cDNA clone incorporating the structural protein coding region of the selected thermostable virus

RNA was extracted from clarified supernatant of passage 9 virus using Trizol (Qiagen) and a Direct-zol purification kit (Zymogen), per the manufacturer’s instructions. The RNA was reverse transcribed and the sequence encompassing residues 784–3888 was amplified using Q5 polymerase (NEB) per the manufacturer’s instructions. Overlap PCR was used to combine the recombinant stabilized virus amplicon with the 5′ UTR derived from our EVA71 infectious clone [34, 35]. The resulting amplicon ligated between *AatII* and *BamHI* within the infectious clone plasmid. The ligated plasmid was transformed in *Escherichia coli* DH5α and plasmids isolated for Sanger sequencing.

### Virus recovery from infectious clone

Virus was recovered as previously described [35], following transfection of *in vitro*-transcribed RNA into HeLa cells. Briefly, the EVA71 infectious clone plasmid was linearized with *Xho*I followed by phenol–chloroform extraction. RNA was synthesized using the RiboMAX T7 express large-scale RNA production system (Promega) and purified using RNA clean and concentrator columns (Zymo Research). RNA was electroporated into HeLa cells within 0.4 mm cuvettes at 260 V for a single pulse of 25 ms using square wave. Cells were transferred to plates and incubated at 37 °C, 5 % CO_2_ in a humidified chamber overnight. Plates were freeze-thawed to enhance viral release from cells, and cellular debris was pelleted at 17 000 r.c.f. for 10 min. The supernatants were collected and samples titred using median tissue culture infectious dose (TCID_50_).

### Viral titration (TCID_50_ and plaque assay)

To titre viral samples by TCID_50_, 1×10^4^ Vero cells were seeded into each well of a 96-well plate in 100 µl of Dulbecco's Modified Eagle Medium (DMEM) supplemented with 2 % FBS. A series of 10-fold serial dilutions were made from viral supernatants in DMEM supplemented with 2 % FBS and 100 µl of each dilution (10^−2^ to 10^−7^) was added to five replicate wells. Plates were returned to a 37 °C, 5 % CO_2_ humidified incubator for 5 days before being fixed and inactivated by the addition of 100 µl per well of 4 % formaldehyde for 30 min. The contents of wells were discarded and residual cells stained with crystal violet solution. Titres were determined using the Reed–Muench method and are expressed as TCID_50_ ml^−1^ [38].

To titre virus by plaque assay, confluent HeLa-seeded six-well plates were inoculated with six 10-fold dilutions of virus supernatant and incubated at 37 °C for 1 h. Virus inoculum was removed and replaced with 2 ml of 0.75 % agarose in DMEM and incubated for 4 days. Wells were fixed with formaldehyde and stained with crystal violet solution. Agarose layers were removed, wells were washed and titres were determined as plaque-forming units (p.f.u.) ml^−1^.

### Virus purification

Virus was grown and purified as described previously [35]. Briefly, HeLa cells were seeded in 6× T175 flasks and incubated in DMEM supplemented with 10 % FBS until cells reached confluence. Flasks were infected with 2×10^6^ TCID_50_ of EVA71 (m.o.i. ~0.1) and incubated for 72 h, at which point complete CPE was apparent. Samples were centrifuged at 4000 r.c.f. for 30 min to pellet cellular debris. Clarified cell culture supernatants were titred and 120 ml (approximately 1.2×10^9^ TCID_50_) precipitated overnight with 8 % (w/v) PEG-8000. Precipitated material was pelleted at 4000 r.c.f. for 30 min, resuspended in 30 ml phosphate-buffered saline (PBS) and clarified at 4000 r.c.f. for 30 min. Virus was subsequently pelleted through a 5 ml 30 % sucrose cushion at 150 000 r.c.f. for 3.5 h in a SW32Ti rotor. Virus pellets were resuspended in 1 ml PBS and clarified at 10 000 r.c.f. for 10 min before layering atop a discontinuous 15–45 % sucrose gradient (15, 20, 25, 30, 45 % sucrose in PBS). Gradients were centrifuged at 50 000 r.c.f. for 12 h at 4 °C and 1 ml fractions were collected manually from the top of the gradient. The presence of viral proteins was determined by Western blot and enzyme-linked immunosorbent assay (ELISA).

Virus samples for antigen conversion assays were concentrated and purified by adding to an Amicon 100 kDa MWCO PES membrane spin concentrator (Merck) and centrifuging at 1000 r.c.f. until the volume had reduced to approximately 100 µl. The flow-through was discarded, and 2.3 ml PBS was added to the top of the column. This process was repeated, and the retained volume was collected and diluted to a final volume of 1.2 ml in PBS to be used in antigen conversion assays by ELISA.

### Western blot

Samples were mixed 1 : 1 (v/v) with 2× Laemmli buffer and denatured at 95 °C for 10 min. Samples were clarified at 17 000 r.c.f. before loading on 12 % (w/v) SDS-PAGE gels using standard protocols. Western blot analysis was carried out as previously described [35]. Briefly, proteins were transferred to PVDF membrane and blocked with 5 % skim milk powder reconstituted in Tris-buffered saline (TBS) supplemented with 0.1 % Tween 20. EVA71 VP0/VP2-reactive proteins were detected using anti-EVA71 VP2 antibody clone 979 (Merck) at 1 : 2000 dilution and anti-mouse IgG HRP conjugate. Blots were developed using chemiluminescent substrate (Promega) and X-ray film.

### ELISA

A sandwich ELISA method was utilized to determine the specific antigenic content of viral samples [35]. Briefly, ELISA plates were coated overnight with polyclonal rabbit anti-EVA71 immune sera at a 1 : 2000 dilution. Samples were added to wells and incubated at 37 °C for 1.5 h. The 16-2-2D scFv fragment or mAb 979 were used to detect NAg and HAg particles, respectively, and were incubated at 37 °C for 1 h. Anti-His HRP was used to detect scFv at a 1 : 1000 dilution (Bio-Rad). Anti-mouse HRP was used at 1 : 1000 dilution (Merck) to detect mAb 979. Samples were detected using OPD and the OD492 nm measured using the Biotek PowerWave XS2 plate reader. Data was graphed using GraphPad Prism software.

## Results

### Selecting thermally stable EVA71

**Fig. 1.**
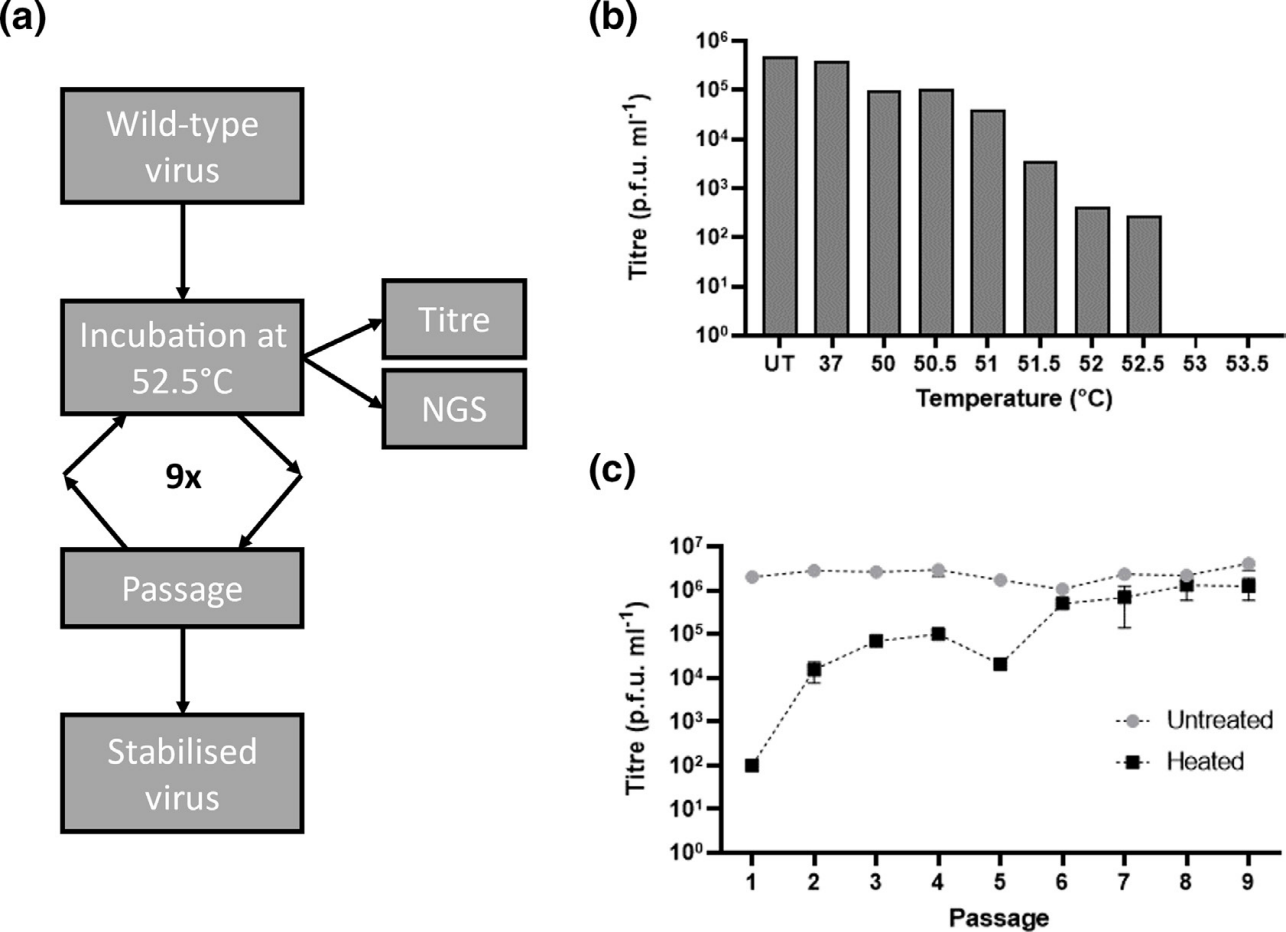
Selection of thermally resistant virus. (**a**) Schematic representation of the experimental process used to select thermally resistant EVA71. (**b**) To determine a suitable incubation temperature for thermal stressing while retaining the ability to recover infectious virus, a series of temperatures were used. Virus stocks at 10^6^ p.f.u. ml^−1^ were incubated at the indicated temperature (*x*-axis) for 30 min and samples were titred by plaque assay, *n*=1. (**c**) To generate a virus population resistant to incubation at the selected temperature (52.5 °C), cycles of heating and recovery were carried out. Heated virus samples were titred by plaque assay and compared to an unheated virus control population, *n*=3 in duplicate, graphed mean±sem.

Before selection of a thermally resistant virus population, the highest temperature from which infectious virus could still be recovered was determined (Fig. 1b). No infectious material remained after incubation at 53 °C and we elected to use 52.5 °C for the subsequent thermal stressing experiments. To this end, an initial stock of 1×10^6^ p.f.u. ml^−1^ wild-type (WT) virus was incubated at 52.5 °C for 30 min and aliquots of the heated sample were titred directly (Fig. 1c) or inoculated onto Vero cells and incubated until full CPE was apparent. The virus recovered from this passage was then used for eight subsequent cycles of heating and recovery (Fig. 1a). Between passages 1–5, heating reduced virus titres by 30–2000-fold compared to the unheated control material. After six cycles or more no significant reduction in titre occurred after heating of the samples (*P*=0.1006; 0.2414; 0.3127; 0.1868, respectively), suggesting that a virus population stably resistant at 52.5 °C had been selected (Fig. 1c). We then determined the genetic changes responsible for the thermal resistance phenotype of this virus population.

### Identification of stabilizing mutations

To determine the genetic changes that may have contributed to the thermally resistant phenotype, samples from unheated virus (passage 9) and heated virus from passages 4, 5, 7, 8 and 9 were subjected to NGS. Several non-coding changes within the 5′ and 3′ UTRs were detected, along with several synonymous changes within the coding sequence of the polyprotein (Table S1). A number of non-synonymous changes were detected within the protein coding region, although two of these were also identified in the untreated WT virus and in other virus samples isolated from the same lineage. We suggest that these changes, VP1-P246A and 3A F12L, may have arisen as a consequence of adaptation to cell culture that occurred after the original virus was sequenced (collected 2017, published 2018 GenBank MG432108.1) (Tables 1 and S2). Consequently, both VP1-P246A and 3A F12L are present in all viruses described here (i.e. unheated, #9 and all infectious clone recovered viruses). Several other non-synonymous changes, including N3225D, I1052T and Q1145L, were detected in earlier passages, but were not seen in samples from passage 7 or later (Table S2). Two additional changes in the non-structural protein coding region were identified; no obvious phenotypic change was associated with the transmembrane domain mutation 3A A66T. The 3C I157V mutation only occurs at low frequency within the population. Therefore, neither mutant was investigated further (Table S2). A total of five non-synonymous mutations were detected in the structural protein coding region of the heated passage 9 virus population, including the putative tissue culture adaptation VP1-P246A (Table 1). Of the additional mutations detected, VP3-I235M was present in >95 % of sequences detected from passage 4 onwards, and by passage 7 VP1-K162I was present in >99 % of sequences. Both VP1-Y116C and VP2-V85L were detected in all heated virus samples sequenced, but at lower frequency than the other non-synonymous mutations present in passage 9 virus (Table 1).

### Location of stabilizing mutations

**Fig. 2.**
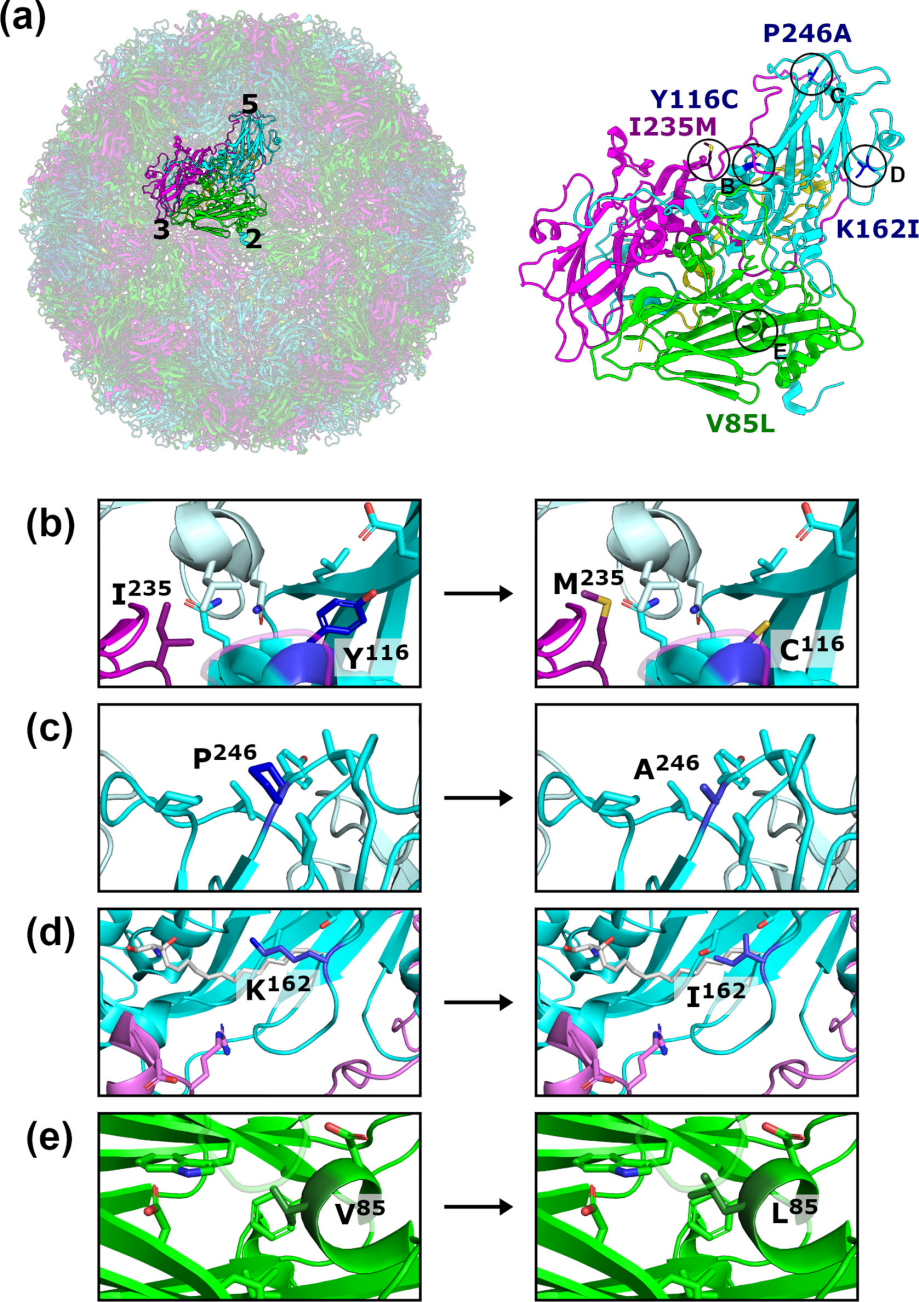
Location of mutations in the stabilized virus population. (**a**) A single protomer highlighted within the context of the full EVA71 capsid (left; PDB: 3VBS, [30]), with icosahedral axes indicated (5, fivefold; 3, threefold; 2, twofold). The protomer is shown in an expanded view (right) with the sites of mutations indicated. VP1, cyan; VP2, green; VP3, magenta; VP4, yellow. (**b–e**) Expanded views showing the sites of mutation indicated in (**a**), with selected side chains shown. Adjacent protomers are indicated by pale colours. In some cases, segments of the backbone were removed or made partially transparent to improve clarity.

The mutations identified within the heated passaged virus are dispersed across the capsid (Fig. 2a). The VP1-P246 residue is located within the VP1 HI loop atop the fivefold mesa (Fig. 2c). VP1-Y116 is present within a helical region between the C- and D-strands, above the canyon and in proximity to the pocket, which often houses a stabilizing lipid molecule (Fig. 2b). The VP3-I235 residue is located in the unstructured region towards the C-terminal end of VP3, which lies above VP1 within the protomer in proximity to VP1-Y116 (Fig. 2b). The VP1-K162 residue is located within the quasi-threefold region formed between adjacent VP1 monomers and the inter-protomer VP3 region (Fig. 2d). VP2-V085 is located between the VP2 C- and D- strands on the external surface of the capsid near the twofold symmetry axis and the inter-pentamer interface (Fig. 2e).

### Verification of thermal resistance

**Fig. 3.**
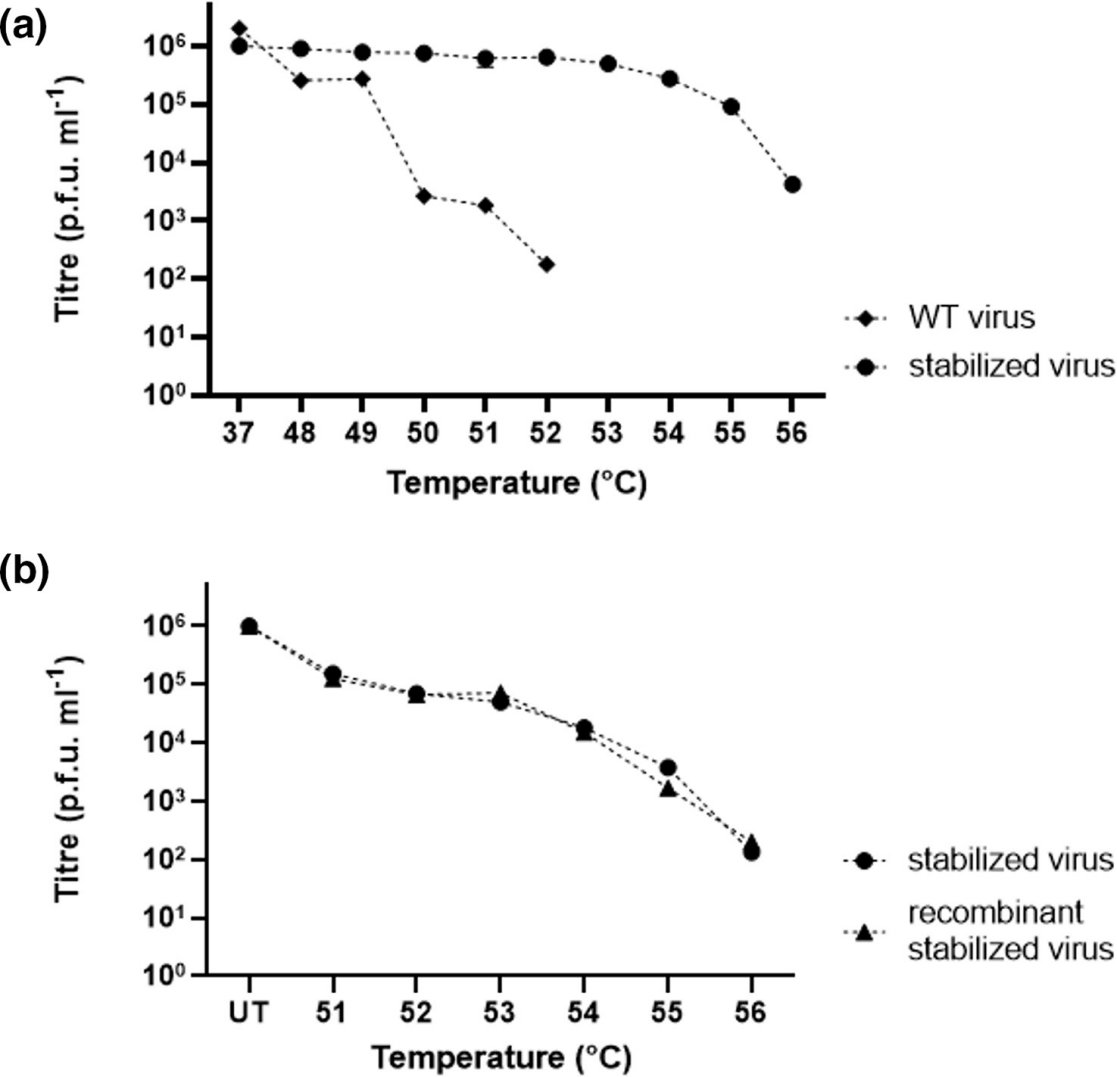
Verification of thermal resistance. (**a**) Characterization of the thermal resistance of WT and stabilized virus populations after incubation at the temperatures indicated along the *x*-axis. (**b**) Comparison of the thermal resistance of stabilized virus and recombinantly produced stabilized virus after incubation at the temperatures indicated along the *x*-axis (UT, untreated). *n*=3 each in duplicate, graphed mean±sem.

The selected virus population was passaged five times in the absence of heating to determine the genetic stability of the thermally resistant phenotype. Sanger sequencing of the resulting virus showed that the mutations present in the P1 region were retained. We also determined the thermal resistance profiles of the virus populations by heating at temperatures up to 56 °C. Similar to the initial selection, WT virus was completely inactivated at temperatures of 53 °C or greater. Interestingly, the stabilized virus population lost only half of the infectious titre at 53 °C and approximately 90 % at 55 °C, while infectious virus was still present after incubation at 56 °C (Fig. 3a).

We subsequently constructed a reverse genetics system encoding the stabilized virus structural coding region to facilitate analysis of the contributions of individual mutations to the thermally resistant phenotype. We isolated and reverse transcribed RNA from the selected thermally resistant virus and amplified the P1 coding region by PCR. We then introduced this DNA into our previously described reverse genetics system by overlap PCR and analysed individual clones [35]. None of the clones sequenced contained the full complement of mutations outlined in Table 1. As multiple clones contained VP3-I235M, VP1-Y116C, VP1-K162I and VP1-P246A, we characterized this combination of mutations in the absence of VP2-V85L. To do so, virus was recovered from T7-transcribed RNA and transfected into L929 cells. The recombinant stabilized virus was then assessed alongside the stabilized virus population for thermal resistance at temperatures ranging from 51–56 °C (Fig. 3b). There was no difference seen in the thermal inactivation phenotype of the stabilized virus population and the recombinant stabilized virus.

### Antigenic characterization of recombinant stabilized virus

**Fig. 4.**
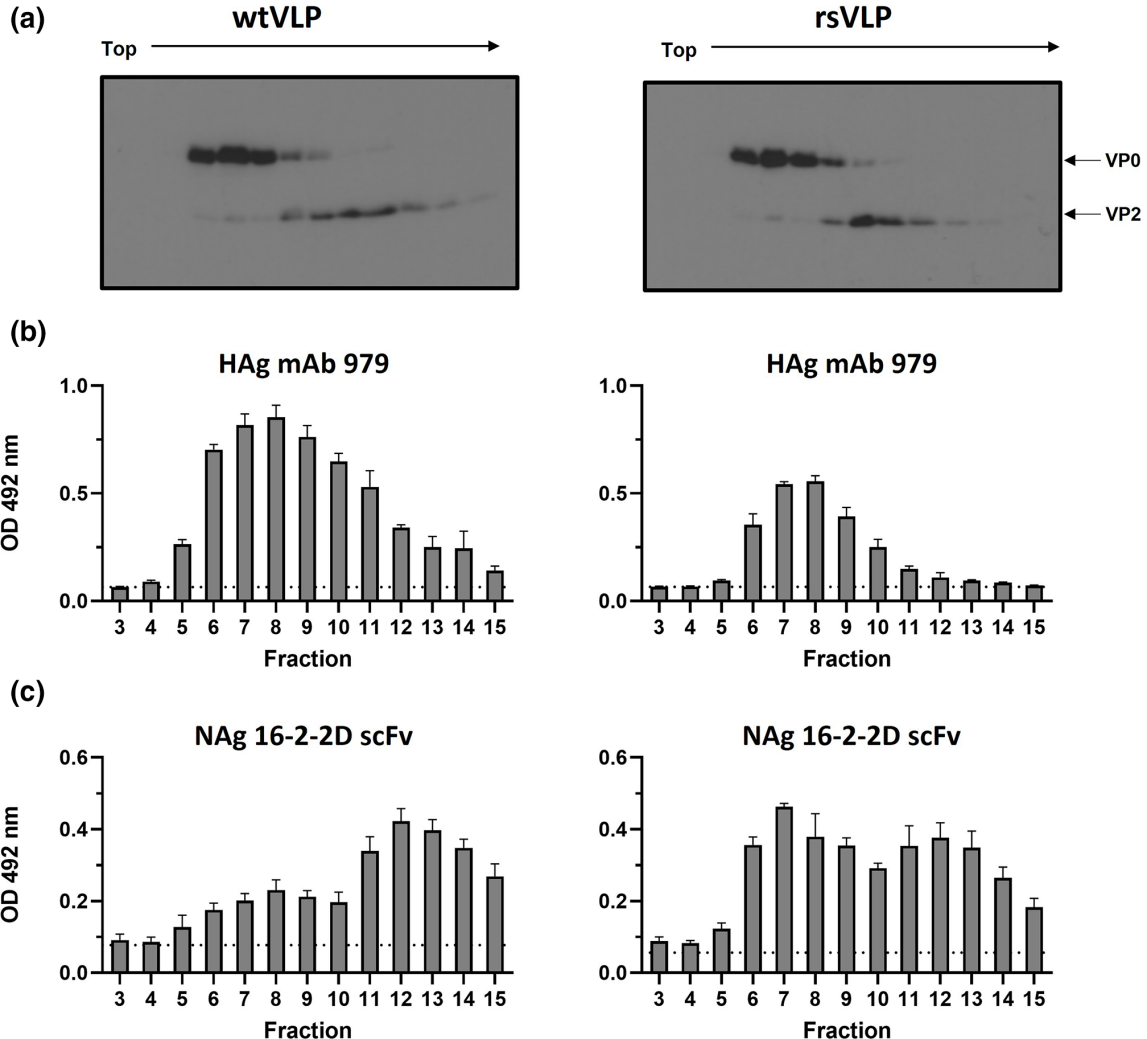
Antigenic form of WT and stabilized ECs and virions. (**a**) Gradient-purified WT and recombinant-stabilized viral samples were assessed for the presence of VP0/VP2 by Western blot using mAb 979, *n*=3 (representative blots shown). (**b, c**) Fractions were assessed by sandwich ELISA for (**b**) expanded particles using mAb 979, (**c**) and native particles using a 16-2-2D scFv. For clarity, fractions are aligned across all panels with wild-type samples on the left and recombinant stabilized samples on the right. For each, *n*=3 in duplicate, graphed mean±sem, dotted line indicates the background of the assay, wells containing no antigen.

We next determined whether the thermal resistance observed for the recombinant stabilized virus extended to any ECs that were produced. To better understand the precise antigenic conformation of these particle types we employed an ELISA specific for the NAg or HAg conformations. Recombinant WT and stabilized viruses were recovered from T7-transcribed RNA, large-scale cultures were generated and material was sedimented through 15–45 % sucrose gradients. Under the conditions used, ECs peaked around fraction 8 and virions around fraction 12. Fractions were assessed for VP0/VP2-reactive proteins by Western blot (Fig. 4a) and for HAg- and NAg-reactive particles by ELISA (Fig. 4b, c, respectively). The WT sample had a profile as expected from our previous work and the published literature [35]. The fractions corresponding to WT ECs were predominantly HAg-reactive, and those corresponding to virions were predominantly NAg-reactive (Fig. 4, left). Interestingly, the ECs derived from stabilized virus cultures were less reactive in the HAg ELISA (Fig. 4b), and had a dominant peak in the NAg ELISA (Fig. 4c), suggesting that the stabilized ECs were in the NAg conformation. As anticipated, virions from WT and recombinant stabilized virus samples reacted predominantly with NAg-specific antibodies (Fig. 4c).

### Antigenic conversion of EC and virus

**Fig. 5.**
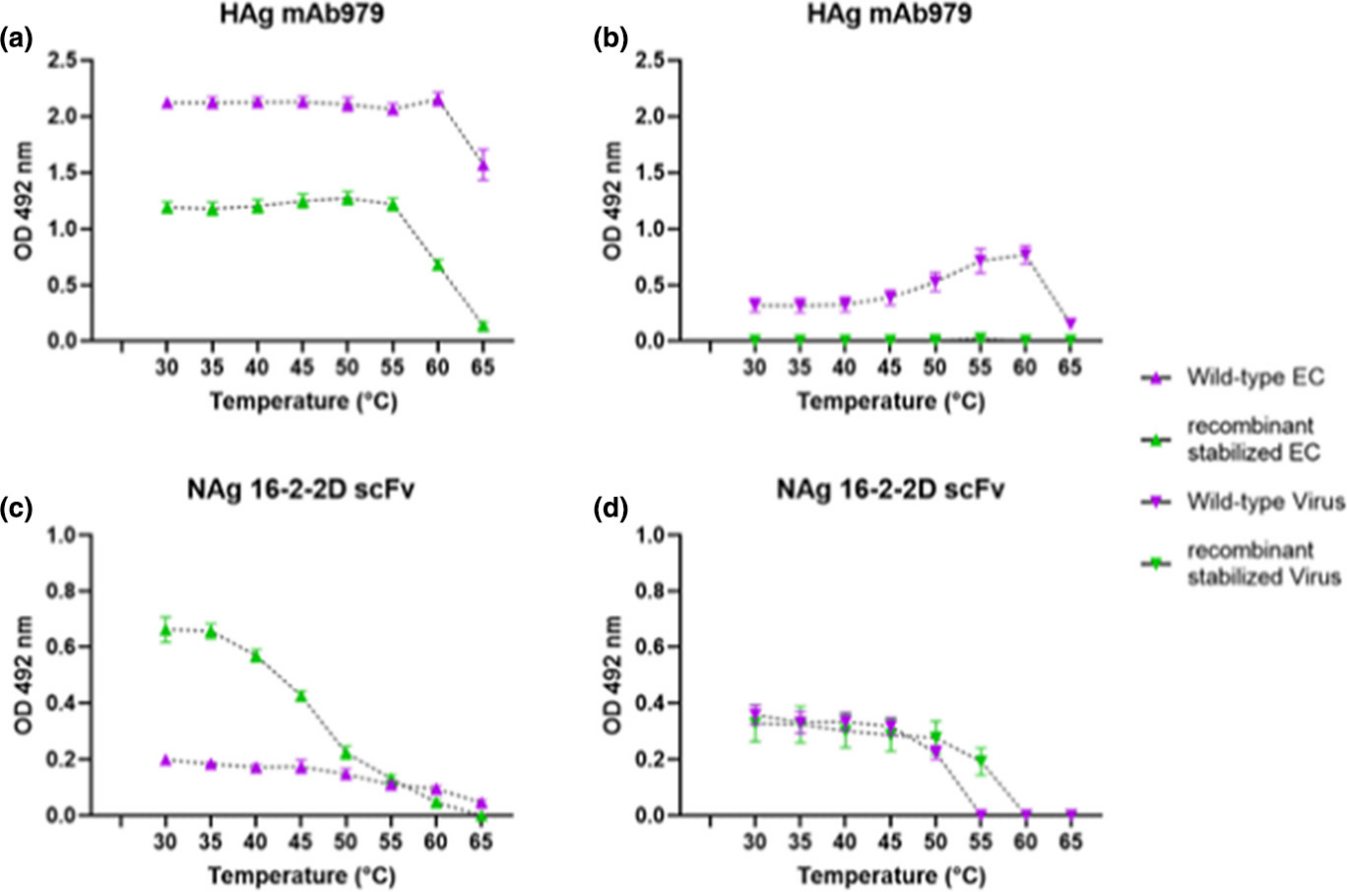
Thermal stability of WT and stabilized ECs and virions. Samples of virus and EC were heated to a range of temperatures to induce antigenic conversion. Graphed HAg-reactive (**a**) EC and (**b**) virus, and NAg-reactive (**c**) EC and (**d**) virus. HAg assays use mAb 979 and NAg assays use the 16-2-2D scFv in the detection phase of a sandwich ELISA. Graphed mean±sem normalized to 2× blank well OD, *n*=3 each in duplicate. WT, wild-type; rs, recombinant stabilized.

To determine the antigenic stability for WT and recombinant stabilized ECs and virions, we firstly reduced the sucrose content of all samples, as sucrose is a thermal protectant [39]. Fractions corresponding to ECs (7–9) and virions (11–13) were pooled from each independent replicate and buffer exchange was carried out using a spin concentrator. The total volume (2.4 ml, 800 µl/fraction) was reduced to approximately 100 µl and reconstituted to 2.4 ml in PBS, and then the volume was again reduced to approximately 100 µl and samples were reconstituted in a final volume of 1.2 ml of PBS. EC and virus samples were subsequently diluted 1 : 2 with PBS and 200 µl aliquots were incubated at temperatures ranging from 30–65 °C using 5 °C increments (±0.2 °C) for 10 min. ECs from both WT and recombinant stabilized samples showed substantial reactivity with mAb 979, suggesting the presence of HAg-reactive particles. This reactivity was lost in the stabilized ECs between 55–65 °C, while WT ECs started to show a reduction in HAg reactivity around 60 °C, suggesting a difference in the stability of the wild-type and recombinant stabilized expanded EC forms (Fig. 5a). Unsurprisingly, the WT and recombinant stabilized virus samples had a lower reactivity with mAb 979, likely due to the presence of NAg particles. Similar quantities of NAg-reactive viral particles were detected in both the WT and recombinant stabilized virus samples based upon reactivity with the 16-2-2D scFv (Fig. 5d). Curiously, at higher temperatures WT virus samples gained HAg reactivity, presumably as a consequence of particle expansion, though we did not see this same change with the recombinant stabilized virus sample (Fig. 5b). Consistent with previous data (Fig. 2a), WT virus lost NAg reactivity between 50–55 °C and recombinant stabilized virus showed a major loss of reactivity between 55–60 °C (Fig. 5d). Critically, the presence of NAg-reactive particles from the recombinant stabilized EC sample suggests that the mutant residues that stabilize the virus also stabilized the NAg form of ECs derived from this mutant sequence (Fig. 5c).

## Discussion

Understanding the functional importance of NAg and HAg conformations for the generation of protective immunity against EVA71 is critical for optimization of vaccine efficacy. Several murine immunization trials have shown that WT VLPs can elicit protective responses, although whether these would be improved or modulated by maximizing the anti-NAg response remains an open question [5–10]. Particles stabilized in the NAg conformation would be a useful resource to address this question. As such, we selected a thermally stable EVA71 virus in the anticipation that ECs derived from this virus would better retain the NAg conformation and be more suitable for future VLP vaccine studies.

We used successive cycles of heating and passage to select for a virus population resistant to thermal inactivation. We used 52.5 °C to select resistant virus as this was the maximum temperature compatible with the recovery of infectious virus (Fig. 1b). After successive cycles the thermal resistance of the recovered virus population increased and by six cycles there was no further loss of titre after heating (Fig. 1c). Two mutations, VP1-P246A and VP3-I235M, appeared at high frequency (>95 %) during early passages but complete thermal resistance correlated with the further acquisition of a third mutation, VP1-K162I (Fig. 1c, Table 1). These data suggest that VP3-I235M, either alone or in combination with the putative tissue culture adaptation mutation VP1-P246A, provide some resistance to heating, which is enhanced by the acquisition of VP1-K162I. In addition to these three mutations the frequency of the mutation VP1-Y116C increased markedly at later passages, suggesting that it may further contribute to stabilization (Table 1). The proportion of VP2-V85L in the population varied throughout the selection series, with no clear relationship to other mutations or thermal resistance (Fig. 1c, Table 1).

With respect to their positions in the capsid structure, the VP2-V85L mutation resides in proximity to the helices at the twofold axis, whereas VP1-Y116C and VP3-I235M lie atop the canyon, and VP1-K162I is located at the q3-fold axis; all of these sites undergo conformational changes resulting from antigenic conversion (Fig. 2) [29–34, 40]. Capsid expansion results in the expulsion of a stabilizing lipid molecule from within the VP1 pocket located at the base of the canyon, essential for viral infectivity [30, 41, 42]. This also correlates with expansion at the twofold axis to allow the externalization of VP4 and the genome [40], and expansion at the q3-fold axis in association with externalization of the VP1 N-terminal region, leading to irreversible conversion from NAg to HAg [34, 40, 42, 43]. Given the proximity of each of our identified mutations to regions associated with antigenic conversion, it is likely that they cooperatively facilitate the increased thermal resistance observed in the stabilized virus population. When the genetic stability of the mutations selected under thermal pressure was investigated by carrying out five further passages in the absence of selection, we saw no reversal in the mutation profile, suggesting that the mutations did not carry a replicative cost under normal passage conditions. The resistance of WT and stabilized virus populations to temperatures above the selection temperature of 52.5 °C was assessed by heating at temperatures up to 56 °C (Fig. 3a). Consistent with our previous observation, the WT virus was completely inactivated at temperatures of 53 °C or greater (Figs 1b and 3a), but the stabilized virus population retained infectivity at 56 °C (Fig. 3a). The thermal resistance phenotype of the selected virus population was replicated in a genetically defined molecular clone comprising the VP3-I235M, VP1-Y116C, VP1-K162I and VP1-P246A mutations (Fig. 3b). This infectious clone did not possess the VP2-V85L mutation, which was detected at moderate frequency in the selected virus population. In addition, the recombinant stabilized virus did not contain any of the additional non-structural mutations detected in the stabilized virus population (Table S2), indicating that this sequence diversity did not contribute to thermal resistance. This contrasts with previous studies of PV that implied that both structural and non-structural mutations can contribute to the stabilization phenotype [44].

The antigenic consequences of the stabilizing mutations were investigated using purified virions (defined by VP2) or ECs (defined by VP0) produced from WT or recombinant stabilized virus, using ELISAs specific for NAg- (16-2-2D scFv) or HAg- (mAb 979)- reactive particles (Fig. 4) [35]. Consistent with our previous observations, WT ECs were predominantly HAg- reactive and WT virions were predominantly NAg-reactive (Fig. 4b, c, respectively) [35]. Interestingly, the ECs derived from the recombinant stabilized sequence were less reactive with HAg-specific antibody (mAb 979) and were highly reactive with the NAg-specific 16-2-2D scFv (Fig. 4b, c, respectively). Together, this indicates that the ECs derived from the recombinant stabilized virus retained NAg reactivity, and thus may be a suitable candidate for the development of stabilized EVA71 VLP vaccines.

The antigenic stabilities of the ECs and virions were investigated by incubating the particles at a range of temperatures and assessing their NAg and HAg reactivity by ELISA. The WT ECs maintained HAg reactivity up to 60 °C, but this decreased at higher temperatures; consistent with this, WT ECs were not reactive in the NAg-specific ELISA (Fig. 5a, c). As expected, the WT virus was detected by NAg ELISA at lower temperatures, although this reactivity was lost between 45–55 °C, consistent with the conversion of the infectious virus to a non-infectious expanded state (Fig. 5c). The loss of NAg reactivity was paralleled by gain of HAg reactivity consistent with conversion of infectious NAg viral particles to the non-infectious HAg conformation (Fig. 5c).

Surprisingly, the recombinant stabilized ECs lost reactivity with mAb 979 at a lower temperature (55 °C) than the equivalent WT sample (Fig. 5a), suggesting alternative expansion pathways for these two particle types. The loss of NAg reactivity for the recombinant stabilized ECs occurred between 35–60 °C with a 50 % conversion temperature around 42 °C (Fig. 5c). Curiously, the loss of NAg-reactive ECs was not paralleled by gain of HAg reactivity. A similar phenomenon was observed for the recombinant stabilized virion, where the loss of NAg reactivity between 55–60 °C does not correlate with a gain in HAg reactivity (Fig. 5).

Thus, although the WT and recombinant stabilized virions have similar NAg reactivity (Fig. 5d), the WT virions convert to a canonical HAg-reactive particle upon heating, the recombinant stabilized virions do not (Fig. 5b, d). The reason for differences in HAg reactivity upon heating may result from the influence of stabilizing mutations on particle expansion at distant sites within the capsid. None of the stabilizing mutations present in the recombinant mutant virus, which lacks the VP2-V85L mutation, are in proximity to the twofold axis, where mAb 979 reacts (VP2 residues 136–150) [45]. The lack of proximity suggests that the mutations are unlikely to directly influence antibody binding. It seems more probable that the conformational changes that lead to expansion in WT particles are not favoured in the presence of the stabilizing mutations. For instance, the HAg reactivity of mAb 979 relies upon the exposure of its epitope, which coincides with a number of structural changes, including the loss of density within the VP1 GH region. It is possible that stabilization at the q3-fold (VP1-K162I) or atop the canyon (VP1-Y116C, VP3-I235M) may influence the conformation of the VP1 GH region, preventing exposure of the mAb 979 epitope. Future studies should include more detailed antigen mapping and structure determination to better understand the mechanism of particle stabilization and why it appears that loss of NAg reactivity does not correlate with a gain in HAg reactivity in stabilized mutant particles.

## Conclusions

We have selected for a combination of mutations that provides NAg stability to ECs. We propose that capsid proteins incorporating this combination of mutations will be ideal candidates for expressing stabilized VLPs in heterologous systems. We further suggest that these particles will facilitate future research to address the NAg/HAg paradigm surrounding EVA71 vaccine efficacy.

## Supplementary Data

Supplementary material 1
