# Supplementary material for: Thermal stabilization of enterovirus A 71 and production of antigenically stabilized empty capsids
Source: J Gen Virol. 2022 Aug 23;103(8):001771. doi: 10.1099/jgv.0.001771 (PMC10019091; doi:10.1099/jgv.0.001771)
Supplement: Supplementary material 1 [file jgv-103-1771-s001.pdf]

Table S1: NGS summary data, non-coding, and synonymous changes relative to the published sequence MG432108.1

CDS	Minimum	Change	CDS Codon	Amino Acid	Average Variant Frequency				
					p4	p5	p7	p8	p9
	147	A -> G				2.4%			
	583	A -> G			8.7%	5.4%			
	600	A -> G			3.3%				
	690	C -> A					2.8%	3.1%	1.85%
	723	G -> A			99.7%	100.0%	100.0%	99.9%	99.90%
	729	A -> G							1.40%
	731	T -> C			100.0%	100.0%	100.0%	99.9%	99.90%
VP4	828	T -> C	27		99.7%	99.8%	99.9%	99.9%	99.90%
VP2	1047	C -> T	31						1.35%
	1248	T -> C	98		6.0%	12.9%			
	1491	C -> T	179						1.25%
	1518	C -> T	188				5.2%	4.5%	2.25%
	1662	C -> T	236		2.4%	6.6%			
VP1	2694	C -> T	84						1.10%
	2724	T -> C	94		4.4%	3.1%			
	3012	C -> T	190		94.9%	96.6%	99.9%	99.7%	99.70%
2A	3354	T -> C	7			4.7%			
	3444	T -> C	37			5.7%	2.7%		1.20%
	3663	C -> T	110		41.7%	60.1%	99.9%	99.7%	99.55%
2B	3933	T -> C	50						2.15%
	4032	T -> C	83		42.5%	60.2%	99.7%	99.5%	99.55%
2C	4134	A -> G	18						1.40%
	4359	A -> G	93		100.0%	100.0%	99.8%	100.0%	99.95%
	4962	C -> T	294		44.4%	62.9%	99.9%	99.7%	99.70%
3C	5586	G -> A	65		3.9%	2.7%			
	5610	A -> G	73			3.0%			
3D	6429	G -> A	163		2.9%				
3'UTR	7341	A -> T			95.6%	95.4%	100.0%	99.7%	99.50%

Table S2: NGS summary data, non-synonymous changes relative to the published sequence MG432108.1

CDS	Minimum	Change	CDS Codon	Amino Acid	Average Variant Frequency				
					p4	p5	p7	p8	p9
VP2	1207	G -> C	85	V -> L	24.3%	14.5%	9.2%	20.6%	43.10%
VP3	2396	A -> G	225	N -> D	21.4%	53.4%			
	2421	A -> G	233	I -> M	95.5%	97.2%	100.0%	99.6%	99.70%
VP1	2597	T -> C	52	I -> T	3.8%	3.1%			1.70%
	2789	A -> G	116	Y -> C	2.3%	2.3%	22.8%	43.5%	69.15%
	2876	A -> T	145	Q -> L	5.2%	3.2%			
	2927	A -> T	162	K -> I	15.6%	21.2%	99.6%	99.9%	99.90%
	3178	C -> G	246	P -> A	95.2%	97.2%	100.0%	99.7%	99.70%
3A	5101	T -> C	12	F -> L	100.0%	100.0%	99.9%	100.0%	99.95%
	5263	G -> A	66	A -> T	95.5%	97.0%	99.9%	99.7%	99.60%
3C	5860	A -> G	157	I -> V			2.4%	4.4%	6.45%
